# Comparative evaluation of two commercial real-time PCR kits (QuantiFast™ and abTES™) for the detection of *Plasmodium knowlesi* and other *Plasmodium* species in Sabah, Malaysia

**DOI:** 10.1186/s12936-020-03379-2

**Published:** 2020-08-27

**Authors:** Nor Afizah Nuin, Angelica F. Tan, Yao Long Lew, Kim A. Piera, Timothy William, Giri S. Rajahram, Jenarun Jelip, Jiloris F. Dony, Rashidah Mohammad, Daniel J. Cooper, Bridget E. Barber, Nicholas M. Anstey, Tock H. Chua, Matthew J. Grigg

**Affiliations:** 1 Faculty of Medicine and Health Sciences, Universiti Malaysia Sabah, Kota Kinabalu, Sabah, Malaysia; 2 Infectious Diseases Society Kota Kinabalu - Menzies School of Health Research Clinical Research Unit, Kota Kinabalu, Sabah, Malaysia; 3 Global and Tropical Health Division, Menzies School of Health Research, Charles Darwin University, Darwin, PO Box 41096, Casuarina, NT 0810 Australia; 4 Gleneagles Hospital, Kota Kinabalu, Sabah, Malaysia; 5 Clinical Research Centre, Queen Elizabeth Hospital, Ministry of Health, Kota Kinabalu, Sabah Malaysia; 6 Ministry of Health, Kuala Lumpur, Malaysia; 7 State Public Health Laboratory, Sabah Department of Health, Kota Kinabalu, Malaysia; 8 QIMR Berghofer Medical Research Institute, Brisbane, QLD Australia

**Keywords:** Zoonotic malaria, *Plasmodium knowlesi*, Real-time polymerase chain reaction, Sabah Malaysia

## Abstract

**Background:**

The monkey parasite *Plasmodium knowlesi* is an emerging public health issue in Southeast Asia. In Sabah, Malaysia, *P. knowlesi* is now the dominant cause of human malaria. Molecular detection methods for *P. knowlesi* are essential for accurate diagnosis and in monitoring progress towards malaria elimination of other *Plasmodium* species. However, recent commercially available PCR malaria kits have unpublished *P. knowlesi* gene targets or have not been evaluated against clinical samples.

**Methods:**

Two real-time PCR methods currently used in Sabah for confirmatory malaria diagnosis and surveillance reporting were evaluated: the QuantiFast™ Multiplex PCR kit (Qiagen, Germany) targeting the *P. knowlesi* 18S SSU rRNA; and the abTES™ Malaria 5 qPCR II kit (AITbiotech, Singapore), with an undisclosed *P. knowlesi* gene target. Diagnostic accuracy was evaluated using 52 *P. knowlesi,* 25 *Plasmodium vivax*, 21 *Plasmodium falciparum*, and 10 *Plasmodium malariae* clinical isolates, and 26 malaria negative controls, and compared against a validated reference nested PCR assay. The limit of detection (LOD) for each PCR method and *Plasmodium* species was also evaluated.

**Results:**

The sensitivity of the QuantiFast™ and abTES™ assays for detecting *P. knowlesi* was comparable at 98.1% (95% CI 89.7–100) and 100% (95% CI 93.2–100), respectively. Specificity of the QuantiFast™ and abTES™ for *P. knowlesi* was high at 98.8% (95% CI 93.4–100) for both assays. The QuantiFast™ assay demonstrated falsely-positive mixed *Plasmodium* species at low parasitaemias in both the primary and LOD analysis. Diagnostic accuracy of both PCR kits for detecting *P. vivax*, *P. falciparum*, and *P. malariae* was comparable to *P. knowlesi*. The abTES™ assay demonstrated a lower LOD for *P. knowlesi* of ≤ 0.125 parasites/µL compared to QuantiFast™ with a LOD of 20 parasites/µL. Hospital microscopy demonstrated a sensitivity of 78.8% (95% CI 65.3–88.9) and specificity of 80.4% (95% CI 67.6–89.8) compared to reference PCR for detecting *P. knowlesi*.

**Conclusion:**

The QuantiFast™ and abTES™ commercial PCR kits performed well for the accurate detection of *P. knowlesi* infections. Although the QuantiFast™ kit is cheaper, the abTES™ kit demonstrated a lower LOD, supporting its use as a second-line referral-laboratory diagnostic tool in Sabah, Malaysia.

## Background

Malaysia has made significant progress towards their World Health Organization (WHO) goal of eliminating human-only malaria by 2020, with no indigenous cases of *Plasmodium falciparum* or *Plasmodium vivax* malaria reported in 2018 [1]. However, the emergence of zoonotic transmission of the monkey parasite *Plasmodium knowlesi* has been less tractable to conventional malaria control efforts [2]. Within Malaysia, *P. knowlesi* is now the most common cause of malaria in humans, accounting for almost all reported malaria cases [1, 3], including over 2000 notifications in the state of Sabah in 2017 [2]. Confirmed *P. knowlesi* human infections have now been reported in all areas of Southeast Asia where the primary reservoir macaque hosts and *Anopheles leucosphyrus* group mosquitoes are present [4–6].

Microscopic assessment of Giemsa-stained blood smears remains the appropriate primary point-of-care method for malaria diagnosis in most *P. knowlesi* endemic countries, including Malaysia [7]. Ideally, differentiation of *Plasmodium* species via microscopy allows initiation of prompt and appropriate treatment and accurate public health reporting [8]. However, well-established limitations in the use of routine diagnostic malaria microscopy include the inability to differentiate between *P. knowlesi* and *P. malariae* due to similar morphology across all life-stages [9, 10]. *Plasmodium knowlesi* is also commonly misidentified as *Plasmodium falciparum* due to similarities in the early ring stage [11], and also with *Plasmodium vivax* in co-endemic areas such as Malaysia [10, 12] and Indonesia [13]. Commercially available pLDH-based rapid diagnostic tests (RDTs) developed for human-only *Plasmodium* species are known to be cross-reactive for *P. knowlesi* epitopes [14]. However, RDTs evaluated for *P. knowlesi* detection to date have demonstrated insufficient sensitivity and specificity to support their use for routine diagnosis [15–18].

Molecular methods are necessary for accurate diagnostic confirmation of *P. knowlesi* and other *Plasmodium* species, and for improved public health malaria surveillance reporting in co-endemic areas in Southeast Asia [11, 19]. In Malaysia, molecular detection methods have been implemented for routine confirmation of all malaria cases since 2014 [7]. In Sabah, the primary molecular detection method at the State Public Health Laboratory (Makmal Kesihatan Awam; MKA) is a multiplex real-time PCR using the QuantiFast™ Multiplex PCR kit (QIAGEN, Germany), which requires addition of previously published primers and probes targeting the SSU rRNA gene of *P. knowlesi* and other human-only *Plasmodium* species [20]. The commercially available abTES™ Malaria 5 qPCR II Kit (AITbiotech, Singapore) is used for subsequent validation of any inconclusive results. However, the gene target is undisclosed, and there are no published data evaluating this assay on clinical *P. knowlesi* samples.

This study aimed to evaluate the diagnostic accuracy and limit of detection of the two commercially available multiplex real-time PCR methods used in routine molecular diagnosis and surveillance reporting of *P. knowlesi* and other human-only *Plasmodium* species in Sabah, Malaysia.

## Methods

### Study details and ethical approval

Patient demographics, clinical data, and blood samples were collected as part of an ongoing prospective malaria study in Sabah, Malaysia. Patients with positive microscopy for malaria and adult healthy controls, subsequently confirmed malaria-negative via reference PCR [21, 22], were enrolled after informed consent was obtained. The study was approved by the national Medical Research Ethics Committee of Malaysia, and Menzies School of Health Research, Australia.

### Blood sample procedures

Venous whole blood was collected from all participants prior to any anti-malarial treatment. Microscopic quantification of *Plasmodium* species parasitaemia was conducted by an experienced research microscopist in Sabah (parasites per microlitre; calculated from the number of parasites per 200 white blood cells on thick blood film, multiplied by the individual patient’s total white cell count [23] obtained from routine hospital laboratory flow cytometry (Full Blood Count results); in the absence of which, an assumption of an average WBC count of 8000/µL of blood was used). EDTA whole blood samples were stored at -80 °C and transported via liquid nitrogen shipper to Darwin, Australia. Genomic DNA was subsequently extracted from 200 µL of whole blood using QIAamp DNA Blood Mini Kits (Cat. No.: 51,106; QIAGEN) according to the manufacturer’s manual, with a final elution volume of 200 µL.

### Detection of *Plasmodium* species using validated reference nested PCR

For *P. knowlesi* detection, a previously validated nested PCR targeting the SSU rRNA gene was utilized, which has a reported specificity of 100% against other *Plasmodium* species infecting humans and/or relevant macaque hosts, and a sensitivity of detection for *P. knowlesi* of 1–10 parasite genomes per microlitre [22]. A separate validated nested PCR was conducted on clinical malaria samples to identify *P. falciparum*, *P. vivax* and *P. malariae* [21]. Samples were then de-identified and randomly assigned onto duplicated 96-well plates for QuantiFast™ and abTES™ PCR evaluation.

### Evaluation of the QuantiFast™ and abTES™ real-time PCR kits

Real-time PCR detection of *Plasmodium* species was performed by laboratory research members blinded to the reference nested PCR results. Both the QuantiFast™ and abTES™ real-time PCR assays were conducted once for each clinical isolate in accordance with the manufacturers’ and Sabah Public Health Laboratory protocols, using the Bio-Rad CFX96 Touch™ PCR machine (Bio-Rad, USA) and duplicated plates of genomic DNA extracted from the same isolates.

QuantiFast™ primer and probe sequences target the *Plasmodium* species-specific 18S SSU rRNA gene for *P. knowlesi* [20] and other human-only *Plasmodium* species [24] (described in Table 1). QuantiFast™ real-time PCR was carried out over two separate reactions due to overlapping emission wavelengths of the reporter probe (carboxyfluorescein; FAM) used to detect both *P. knowlesi* and *P. malariae*. Each final reaction volume of 25 µL consisted of 2 µL DNA template, 12.5 µL master mix, 0.5 µL ROX solution and 1.25 µL 10 × *Plasmodium* species-specific primer–probe mixture. In the first QuantiFast™ reaction, a monoplex real-time PCR amplification was performed to detect *P. knowlesi*. Cycling conditions consisted of: initial Taq activation step at 95 °C for 5 min, followed by 45 two-step cycles of denaturation at 95 °C for 30 s, and annealing/extension at 60 °C for 30 s. In the second QuantiFast™ reaction, a triplex amplification was conducted to detect *P. falciparum*, *P. vivax*, and *P. malariae*. Cycling conditions included: Taq activation at 95 °C for 5 min, followed by 45 cycles of denaturation at 95 °C for 45 s, and annealing/extension at 60 °C for 45 s.

**Table 1:** Primers and probe sequences used for QuantiFast™ real-time PCR detection of *Plasmodium* species [20, 24] and nested PCR detection of *P. knowlesi* [22] in this study

Species	Primer or probe	Sequence (5′–3′)^a^
*P. knowlesi*		
Forward	Plasmo1	GTTAAGGGAGTGAAGACGATCAGA
Reverse	Plasmo2	AACCCAAAGACTTTGATTTCTCATAA
RT-probe	Pkprobe	FAM-CTCTCCGGAGATTAGAACTCTTAGATTGCT-BHQ-1
*P. falciparum*^b^		
Forward	Fal-F	CCGACTAGGTGTTGGATGAAAGTGTTAA
RT-probe	Falcprobe	Cy5-AGCAATCTAAAAGTCACCTCGAAAGATGACT-BHQ-1
*P. vivax*^b^		
Forward	Viv-F	CCGACTAGGCTTTGGATGAAAGATTTTA
RT-probe	Vivprobe	TR-AGCAATCTAAGAATAAACTCCGAAGAGAAAATTCT-BHQ-2
*P. malariae*^b^		
Forward	Mal-F	CCGACTAGGTGTTGGATGATAGAGTAAA
RT-probe	Malaprobe	FAM-CTATCTAAAAGAAACACTCAT-MGBNFQ
Validated reference PCR for *P. knowlesi* detection		
Nest 1; Forward	PkF1160	GATGCCTCCGCGTATCGAC
Nest 1; Reverse	PKR1150	GAGTTCTAATCTCCGGAGAGAAAAGA
Nest 2; Forward	PKF1140	GATTCATCTATTAAAAATTTGCTTC
Nest 2; Reverse	PKR1150	*Same as reverse primer used in Nest 1*

^a^MGBNFQ: minor groove binding non-fluorescent quencher; BHQ: black hole quencher; Cy5: cyanine; FAM: carboxyfluorescein; TR: Texas Red

^b^The Plasmo2 reverse primer was also used for *P. falciparum*, *P. vivax* and *P. malariae*

The abTES™ reaction was performed using the abTES™ Malaria 5 qPCR II kit, which came with primer–probe mixtures and positive controls for detection of *P. knowlesi* and four human-only *Plasmodium* species. The reaction mixture contained 5.0 µL template DNA, 6 µL reaction mix, 2 µL of primer–probe mix, with the final volume adjusted to 25 µL with nuclease-free water. Cycling conditions included: *Taq* activation at 95 °C for 2 min, followed by 45 cycles of amplification at 95 °C for 5 s, and 60 °C for 20 s. The detection channels used were QUASAR 705 (*P. knowlesi*), FAM (*P. falciparum*), ROX (*P. vivax*), and HEX (*P. malariae*), with fluorescence measured at the end of each cycle of amplification.

For both QuantiFast™ and abTES™ reactions, samples were considered positive by determining the threshold cycle number (C_T_) at which normalized reporter dye emission raised above background noise. If the fluorescent signal did not rise above the threshold at 40 cycles (C_T_ 40), the sample was considered negative. Due to the lack of endemic *Plasmodium ovale wallikeri* or *Plasmodium ovale curtisi* in Malaysia, detection of these *Plasmodium* species was not evaluated.

### Limit of detection determination for each PCR method and *Plasmodium* species

The two PCR kits were also systematically evaluated for their respective parasite count limit of detection (LOD) for *P. knowlesi*, *P. falciparum* and *P. vivax* samples. In brief, a single clinical standard isolate for each *Plasmodium* species with high-quality research microscopic enumeration of parasite counts was utilized after reference PCR-confirmation. Individual quantified whole blood samples were then diluted using fresh malaria-negative blood in order to achieve pre-determined parasite counts, before subsequent genomic DNA extraction. The standardized concentrations included in the final analysis for each isolate were: 200, 20, 2, 0.5, 0.25, and 0.125 parasites/µL. The same clinical isolates at each parasite count concentration were used for both PCR detection kits, enabling a direct comparison of the final measured LOD. Assays were conducted in triplicate at each parasite count concentration, with the LOD defined as the lowest concentration at which a positive result was recorded for all 3 replicates.

### Statistical analysis

All statistical analyses were performed using STATA v16 (TX, USA). The primary analysis compared the diagnostic accuracy of the QuantiFast™ and abTES™ kits to detect each *Plasmodium* species infection against the reference PCR result. Diagnostic tests for sensitivity, specificity, and positive and negative predictive values were evaluated [25], as defined below using the number of true positive (TP), false negative (FN), false positive (FP) and true negative (TN) results:

*Sensitivity*: proportion of those with the malaria species correctly identified = TP/(TP + FN)

*Specificity*: proportion of those without the malaria species correctly identified = TN/(FP + TN)

*Area under the Receiver Operating Characteristic (ROC) curve*: average of sensitivity and specificity

*Positive Predictive Value*: probability of the patient having malaria following a positive test = TP/(TP + FP)

*Negative Predictive Value*: probability of the patient having malaria following a negative test = TN/(TN + FN).

Exact binomial confidence intervals of 95% for each of the above diagnostic metrics were calculated and reported. Dependent comparisons between the separate PCR kits diagnostic performance on the same patient’s sample (e.g. QuantiFast™ versus abTES™ for *P. knowlesi* samples) were conducted using McNemar’s test [26]. Independent comparisons using the same PCR test between patients with different *Plasmodium* species infections were conducted using Fisher’s exact test for equality of proportions (e.g. QuantiFast™ performance for detecting *P. knowlesi* versus *P. vivax*). Overall PCR assay performance was compared by testing equality of the receiver operating characteristic (ROC) areas. Age and parasitaemia were compared across *Plasmodium* species results using one-way ANOVA after transformation to a normal distribution, followed by Student’s *t* test for pairwise comparisons; gender was compared using Chi squared test.

## Results

A total of 134 samples collected from Dec 2012 to Feb 2016 were included in the primary analysis evaluating the performance of the real-time PCR detection methods, including: 52 *P. knowlesi*, 21 *P. falciparum*, 25 *P. vivax*, and 10 *P. malariae* monoinfections, and 26 malaria-negative controls. The median age for those with *P. knowlesi* was 35 years (range 25–47) which was higher than patients infected with other *Plasmodium* species (p < 0.001) (Table 2). *Plasmodium knowlesi*-infected patients had a geometric mean parasite count of 9435/µL, similar to those with *P. vivax* (p = 0.99), higher than the 1023 parasites/µL seen for *P. malariae* (p < 0.001), and lower than the 24,631 parasites/µL for *P. falciparum* (p = 0.002). Performance of the screening hospital microscopy result for detecting *P. knowlesi* compared to the reference PCR demonstrated a sensitivity of 78.8% (95% CI 65.3–88.9) and specificity of 80.4% (95% CI 67.6–89.8), with 21.2% of samples diagnosed as *P. malariae*.

**Table 2:** Patient demographic details, clinical data, and diagnostic accuracy of screening hospital microscopy

Species (no. of patients tested), or parameter	*P. knowlesi* (52)	*P. vivax* (25)	*P. falciparum* (21)	*P. malariae* (10)	Negative controls (26)	p-value
Age, median years (range)	35 (25, 47)	18 (9, 34)	16 (10, 39)	14 (7, 23)	44 (22, 76)	**< ***0.001*^**a**^
Sex, *n* male (%)	43 (83)	18 (72)	20 (95)	7 (70)	12 (46)	0.001
Parasitaemia, geometric mean parasites/µL, (95% CI), [range]	9435 (7320–12,161) [138–35,873]	9411 (7004–12,645) [625–36,248]	24,631 (14,406–42,115) [1074–297,000]	1023 (586–1787) [224–3056]	–	**< ***0.001*^b^
Hospital microscopy^c^						
Sensitivity^d^, TP/TP + FN; % (95% CI)	41/52; 78.8 (65.3, 88.9)	23/25; 92.0 (74.0, 99.0)	17/21; 81.0 (58.1, 94.6)	0/10; 0 (0, 30.8)	–	< 0.001
Specificity, TN/TN + FP; % (95% CI)	45/56; 80.4 (67.6, 89.8)	83/83; 100 (95.7, 100)	86/87; 98.9 (93.8, 100)	87/98; 88.8 (80.8, 94.3)	–	< 0.001

^a^*P. knowlesi* compared to: *P. vivax* (t = 3.3, df = 39, p = 0.002); *P. falciparum* (t = 2.6, df = 29, p = 0.02); *P. malariae* (t = 4.8, df = 15, p < 0.001); *P. knowlesi* against negative controls (t = 2.1, df = 54, p = 0.04)

^b^*P. knowlesi* compared to: *P. vivax* (t = 0.1, df = 59, p = 0.99); *P. falciparum* (t = 3.3, df = 30, p = 0.002); *P. malariae* (t = 8.0, df = 14, p < 0.001)

^c^Hospital microscopy compared against reference PCR

^d^2 *P. vivax* monoinfections misidentified as *P. knowlesi*; 4 *P. falciparum* infections misidentified as mixed *P. knowlesi/P. falciparum;* 10 *P. malariae* infections misidentified as *P. knowlesi*

### Diagnostic accuracy of the QuantiFast™ and abTES™ PCR detection methods for *P. knowlesi*

The 52 clinical samples with confirmed *P. knowlesi* monoinfections were evaluated by both the QuantiFast™ and abTES™ PCR methods, and compared against the 83 *P. knowlesi* negative samples (other *Plasmodium* species and malaria negative controls combined) (Table 3). The sensitivity of the QuantiFast™ assay to detect *P. knowlesi* was 98.1% (95% CI 89.7–100), with 51 out of 52 samples recording positive results. The single false negative result was recorded from a 59-year old female patient with a parasitaemia of 12,968 parasites/µL. Specificity of the QuantiFast™ test was 98.8% (95% CI 93.4–100), with a single false positive result (mixed *P. knowlesi/P. malariae*) reported for a *P. malariae* monoinfection on reference PCR with a low parasite count of 224 parasites/µL. QuantiFast™ demonstrated high diagnostic accuracy with an ROC curve area of 0.98 (95% CI 0.96–1.0), a PPV of 98.1% (95% CI 89.7–100) and a NPV of 98.8% (95% CI 93.4–100).

**Table 3:** Diagnostic performance of the QuantiFast™ and abTES™ PCR for detecting *P. knowlesi*

	QuantiFast™	abTES™	p-value
Sensitivity, TP/(TP + FN); % (95% CI)	51/52^a^; 98.1 (89.7, 100)	52/52; 100 (93.2, 100)	0.32
Specificity, TN/(TN + FP); % (95% CI)	81/82^b^; 98.8 (93.4, 100)	81/82^c^; 98.8 (93.4, 100)	1.0
ROC area, (95% CI)	0.98 (0.96, 1)	0.99 (0.98, 1)	0.46
PPV, TP/(TP + FP); % (95% CI)	51/52; 98.1 (89.7, 100)	52/53; 98.1 (89.9, 100)	0.56
NPV, TN/(TN + FN); % (95% CI)	81/82; 98.8 (93.4, 100)	81/81; 100 (95.5, 100)	0.56

*TP* true positive, *FN* false negative, *TN* true negative, *FP* false positive, *ROC* receiver operating characteristic, *PPV* positive predictive value, *NPV* negative predictive value

^a^A false negative result was recorded (negative for all Plasmodium species) for a *P. knowlesi* sample

^b^A false positive result was recorded (a mixed *P. knowlesi*/*P. malariae*) for a *P. malariae* monoinfection

^c^A false positive result was recorded (*P. knowlesi*) for a malaria negative control

In contrast, the abTES™ PCR was positive for all 52 *P. knowlesi* clinical samples, resulting in 100% sensitivity (95% CI 93.2–100). The specificity of the abTES™ assay was the same as seen for QuantiFast™ at 98.8% (95% CI 93.4–100), with a single false positive result recorded as *P. knowlesi* from a malaria-negative healthy control. The overall test accuracy was excellent, with a ROC curve area of 0.99 (95% CI 0.98–1.0), and a high PPV of 98.1% (95% CI 89.9–100) and NPV of 100% (95% CI 95.5–100). There were no statistically significant differences in any diagnostic metric between QuantiFast™ and abTES™.

### Evaluation of QuantiFast™ and abTES™ for detecting *P. vivax*, *P. falciparum* and *P. malariae*

The detection of *P. vivax* was identical for both PCR methods, with a sensitivity for both of 100% (95% CI 86.3–100), and a specificity of 100% (95% CI 96.7–100) (Table 4). Both PCR methods had 100% sensitivity and specificity for detecting *P. falciparum*, including at the lowest tested parasitaemia of 1074 parasites/µL. For detection of *P. malariae*, the sensitivity of the QuantiFast™ was 90.0% (95% CI 55.5–99.7) due to a single false-negative result. In comparison the abTES™ correctly identified all 10 *P. malariae* infected samples, resulting in a sensitivity of 100% (95% CI 69.2–100); p = 0.32. The specificity for both PCR methods was 100% (95% CI 97.1–100).

**Table 4:** Evaluation of (a) QuantiFast™ and (b) abTES™ for *P. knowlesi* compared to other *Plasmodium* species

	*P. knowlesi*		*P. vivax*		p-val (*Pk* vs *Pv*)	*P. falciparum*		p-val (*Pk* vs *Pf*)	*P. malariae*		p-val (*Pk* vs *Pm*)
(a) *QuantiFast™*											
PCR reference	+	−	+	−		+	−		+	−	
Test positive +	51	1	25	0		21	0		9	1*	
Test negative −	1	81	0	109		0	113		0	124	
Sensitivity, % (95% CI)	98.1 (89.7, 100)		100 (86.3, 100)		0.49	100 (83.9, 100)		0.52	90.0 (55.5, 99.7)		0.20
Specificity, % (95% CI)	98.8 (93.4, 100)		100 (96.7, 100)		0.25	100 (96.8, 100)		0.24	100 (97.1, 100)		0.22
ROC area, (95% CI)	0.98 (0.96, 1)		1 (1, 1)		0.15	1 (1, 1)		0.15	0.95 (0.85, 1)		0.57
PPV, % (95% CI)	98.1 (89.7, 100)		100 (86.3, 100)		0.49	100 (83.9, 100)		0.52	100 (66.4, 100)		0.68
NPV, % (95% CI)	98.8 (93.4, 100)		100 (96.7, 100)		0.25	100 (96.8, 100)		0.24	99.2 (95.6, 100)		0.77
(b) *abTES™*											
PCR reference	+	−	+	−		+	−		+	−	
Test positive +	52	0	25	0		21	0		10	0	
Test negative −	1	81	0	109		0	113		0	124	
Sensitivity, % (95% CI)	100 (93.2, 100)		100 (86.3, 100)		1.0	100 (83.9, 100)		1.0	100 (69.2, 100)		1.0
Specificity, % (95% CI)	98.8 (93.4, 100)		100 (96.7, 100)		0.25	100 (96.8, 100)		0.24	100 (97.1, 100)		0.22
ROC area, (95% CI)	0.99 (0.98, 1)		1 (1, 1)		0.31	1 (1, 1)		0.31	1 (1, 1)		0.31
PPV, % (95% CI)	98.1 (89.9, 100)		100 (86.3, 100)		0.49	100 (83.9, 100)		0.52	100 (69.2, 100)		0.66
NPV, % (95% CI)	100 (95.5, 100)		100 (96.7, 100)		1.0	100 (96.8, 100)		1.0	100 (97.1, 100)		1.0

*Includes a mixed *P. malariae/P. knowlesi* result which was false negative for a *P. malariae* monoinfection on reference PCR

QuantiFast™ and abTES™ performed equally well for detecting other *Plasmodium* species compared to *P. knowlesi*, with no statistically significant differences for any measure of diagnostic accuracy (Table 4). There were also no differences in diagnostic accuracy when comparing QuantiFast™ versus abTES™ within each separate *Plasmodium* species group (Table 5).

**Table 5:** Comparison of test performances between QuantiFast™ and abTES™ for detection of *P. knowlesi*, *P. vivax*, *P. falciparum* and *P. malariae*

	Sensitivity (95% CI min, max)			Specificity (95% CI min, max)			ROC area (95% CI min, max)			Positive predictive value (PPV) (95% CI min, max)			Negative predictive value (NPV) (95% CI min, max)		
	QuantiFast™	abTES™	p-val	QuantiFast™	abTES™	p-val	QuantiFast™	abTES™	p-val	QuantiFast™	abTES™	p-val	QuantiFast™	abTES™	p-val
*P. knowlesi* (n = 52)	98.1 (89.7, 100)	100 (93.2, 100)	0.32	98.8 (93.5, 100)	98.8 (93.5, 100)	1.0	0.98 (0.96, 1)	0.99 (0.98, 1)	0.46	98.1 (89.7, 100)	98.1 (89.9, 100)	0.56	98.8 (93.5, 100)	100 (95.6, 100)	0.56
*P. vivax* (n = 25)	100 (86.3, 100)	100 (86.3, 100)	1.0	100 (96.7, 100)	100 (96.7, 100)	1.0	1.0 (1, 1)	1.0 (1, 1)	1.0	100 (86.3, 100)	100 (86.3, 100)	1.0	100 (96.7, 100)	100 (96.7, 100)	1.0
*P. falciparum* (n = 21)	100 (83.9, 100)	100 (83.9, 100)	1.0	100 (96.8, 100)	100 (96.8, 100)	1.0	1 (1, 1)	1 (1, 1)	1.0	100 (83.9, 100)	100 (83.9, 100)	1.0	100 (96.8, 100)	100 (96.8, 100)	1.0
*P. malariae* (n = 10)	90.0 (55.5, 99.7)	100 (69.2, 100)	0.32	100 (97.1, 100)	100 (97.1, 100)	1.0	0.95 (0.85, 1)	1 (1, 1)	0.32	100 (66.4, 100)	100 (69.2, 100)	0.32	99.2 (95.7, 100)	100 (97.1, 100)	0.32

### Limit of detection evaluation

**Fig. 1:**
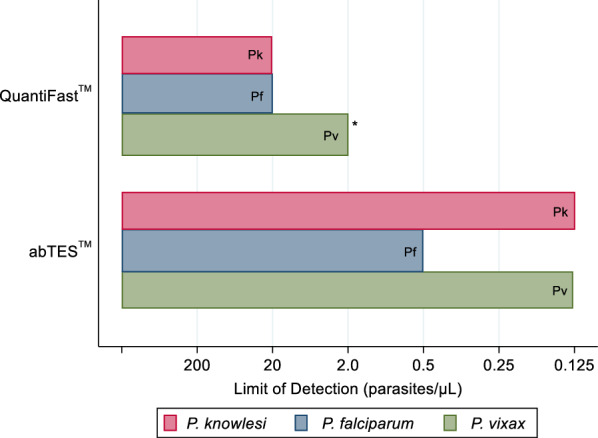
Limit of detection of the QuantiFast™ and abTES™ assays for *P. knowlesi*, *P. falciparum* and *P. vivax.* Limit of detection was defined as the lowest pre-determined parasitaemia required for consistent 100% detection rate based on three replicates at each parasite count level. *False-positive results were recorded (mixed *P. falciparum*/*P. vivax*) for *P. vivax* monoinfections at 2 and 20 parasites/µL

The abTES™ PCR method was shown to have a lower LOD than QuantiFast™ for all *Plasmodium* species, including a documented LOD at the lowest level of dilution evaluated of 0.125 parasites/µL for both *P. knowlesi* and *P. vivax*, and 0.5 parasites/µL for *P. falciparum* (Fig. 1).

The QuantiFast™ method demonstrated a LOD of 20 parasites/µL for both *P. knowlesi* and *P. falciparum*. For *P. vivax*, although 3 out of 3 replicates were positive at 0.25 parasites/µL, a single replicate remained negative at 0.5 parasites/µL, resulting in a final LOD of 2.0 parasites/µL. The evaluation of *P. vivax* for QuantiFast™ was also complicated by single replicates reported as mixed *P. vivax*/*P. falciparum* at both 2 and 20 parasites/µL.

## Discussion

Both the QuantiFast™ and abTES™ real-time PCR assays evaluated in this study demonstrated high diagnostic accuracy in detecting *P. knowlesi* and other *Plasmodium* species monoinfections when compared against the reference nested PCR. The experimentally determined LOD for *P. knowlesi* of 20 parasites/µL for QuantiFast™ and ≤ 0.125 parasites/µL for abTES™ were also both below typical microscopic malaria detection limits [8], further highlighting their utility for confirmatory referral-laboratory diagnostic and surveillance purposes when conducted on point-of-care malaria microscopy-positive samples. Although results suggested a trend towards superior performance of the abTES™ assay and the single reaction required to conduct this assay using current protocols has logistical advantages, abTES™ (~ USD $15.40 per reaction) has a threefold higher cost than QuantiFast™ (~ USD $5.19 per reaction). Therefore, this study supports the use of the current malaria diagnostic and surveillance algorithm used by the Sabah State Public Health Laboratory in an area approaching elimination of human-only *Plasmodium* species, whereby all microscopy positive malaria patients are tested initially using the QuantiFast™ assay, with abTES™ used for any subsequent negative or mixed *Plasmodium* species infection results.

Multiple sensitive molecular methods for *P. knowlesi* detection have been published to date including nested [4, 22], single-step [27–29], and real-time PCR [30, 31], and loop-mediated isothermal amplification (LAMP) [32–35]. Although these molecular methods are directed against a range of different *P. knowlesi* gene targets, their reported detection limits are all below that of routine microscopic examination of malaria blood films. Real-time PCR has a number of advantages compared to conventional nested PCR, including the ability to simultaneously detect multiple *Plasmodium* species in a single amplification round, with higher throughput potential, and does not require manual quantification of end-points using gel electrophoresis [20]. However, real-time PCR requires expensive customized hydrolysis probes in addition to the pre-selected primers for common targets such as *P. knowlesi*-specific 18S SSU rRNA [36], as utilized by the QuantiFast™ assay. This validated gene target is also commonly used for both real-time and nested PCR methods for other *Plasmodium* species differentiation [37], due to a unique *Plasmodium* genus core sequence and a separate highly conserved *Plasmodium* species-specific region [38], resulting in improved diagnostic specificity. In this study, the target gene sequences of the abTES™ method are undisclosed, however gel electrophoresis visualization of the respective *Plasmodium* species-specific PCR products showed amplicon lengths consistent with standard *Plasmodium* 18S SSU rRNA gene sequences [21].

The use of confirmatory molecular detection methods have enabled accurate reporting of malaria trends in Malaysia demonstrating increasing *P. knowlesi* incidence [3], and have also provided reliable data on national and sub-national progress towards achieving elimination of other human-only *Plasmodium* species [2]. In other co-endemic settings in Southeast Asia, the incorporation of *P. knowlesi* detection into existing nucleic acid-based detection protocols would improve their use in targeted malaria surveillance strategies and accuracy of case reporting, particularly on those reported as *P. malariae* or indeterminate *Plasmodium* species infections from point-of-care microscopy [13, 19]. Additionally, this would allow improved understanding of regional diversity in the epidemiology of *P. knowlesi* transmission, and assist in the design of appropriate local preventive public health interventions. The use of molecular detection methods have also enabled evaluation and improvements of local treatment guidelines for knowlesi malaria, including recommending early intravenous artesunate for those with parasitaemia ≥ 20,000/µL [39], and artemisinin-based combination therapy (ACT) for uncomplicated disease in Malaysia [40, 41]. Finally, molecular detection methods may aid surveillance for another zoonotic monkey parasite, *Plasmodium cynomolgi*, with increasing case-reports highlighting spill-over infections occurring in humans in Sabah [42], Peninsular Malaysia [43] and Cambodia [44]. *Plasmodium cynomolgi* is morphologically similar to *P. vivax* on microscopic blood film evaluation [45], and due to being closely genetically related to *P. vivax*, previous PCR detection methods have also demonstrated cross-reactivity between these *Plasmodium* species [44]. This may have implications for accuracy of *P. vivax* case reporting, and potential underestimation of *P. cynomolgi* incidence in Southeast Asia [19].

The QuantiFast™ assay evaluated in this study is currently favoured by the Sabah State Public Health Laboratory due to its lower cost compared to abTES™, despite requiring two reactions; i.e. one monoplex and one triplex for each clinical isolate. The lengthier run time for QuantiFast™ is further compounded by a more tedious sample preparation. However, the technical issue of requiring two reactions per sample for the current QuantiFast™ laboratory protocol could be overcome by changing one of the FAM reporter dyes currently used for both *P. malariae* or *P. knowlesi* probes (Table 1), thus ensuring non-overlapping of emission wavelengths across four probes. As this study replicated the current public health surveillance protocol, the development of a new probe using a fourth colour would require additional validation. Future development of the QuantiFast™ method could, therefore, result in a single quadruplex reaction, further reducing operational cost and time, and minimization of errors, which would ideally include detection of *P. cynomolgi* if an appropriately validated probe becomes available.

One limitation of this study related to the secondary LOD analysis, where the lowest pre-selected parasitaemia (0.125 parasites/µL) remained above the actual limit of detection for *P. knowlesi* and *P. vivax* when using the abTES™ method. Difficulties with the conduct of PCR diagnostics in reference or research laboratory settings are evident for many pathogens, and may not reflect the ideal technical accuracy of the diagnostic assay. It is also not possible to preclude the possibility of abTES™ real-time PCR having greater sensitivity for detection of *P. knowlesi* as compared to the reference PCR [22]. Although unlikely, the single positive result for abTES recorded from a healthy control in an endemic area may have been a genuine asymptomatic submicroscopic *P. knowlesi* infection. A single *P. knowlesi* sample with a parasite count of 12,968/µL, a level well above the documented LOD, was found to be falsely negative on the QuantiFast™ assay, but positive with abTES™, which may have indicated a possible error during sample loading. The specificity of the QuantiFast™ assay for *P. knowlesi* detection was reduced by a single false-positive mixed *P. knowlesi/P. malariae* result for a *P. malariae* monoinfection of 224 parasites/µL, the corresponding abTES™ result was positive for *P. malariae* only. This anomaly may have been caused by unintended annealing of *P. knowlesi*-specific probes onto *P. malariae* genomic DNA [46]; a plausible scenario given primers used for the QuantiFast™ *P. knowlesi* monoplex are not *Plasmodium* species-specific. The QuantiFast™ assay also demonstrated a false-positive mixed *P. falciparum/P. vivax* result from a *P. vivax* monoinfection during the LOD analysis at low-level parasitaemia (2 and 20 parasites/µL). These findings imply that in routine surveillance in Sabah, mixed *Plasmodium* species infections using QuantiFast™ may require further validation.

## Conclusion

The QuantiFast™ and abTES™ methods for detection of *P. knowlesi* infections both performed to an appropriately high standard in the primary evaluation of this study, supporting their continued usage for confirmatory malaria diagnosis and accurate malaria surveillance reporting in Sabah, Malaysia. The QuantiFast™ kit is a cost-effective initial method, with the abTES™ kit appropriate for second-line confirmation of negative or mixed *Plasmodium* species infections due to potentially improved sensitivity and specificity.

## Data Availability

The datasets used and/or analysed during the current study are available from the corresponding authors upon reasonable request.
